# Long-term relative survival with and without radioiodine in patients with low-risk thyroid cancer: a SEER based analysis of histologic subtypes and risk factors

**DOI:** 10.1007/s00259-026-07888-1

**Published:** 2026-05-04

**Authors:** Henning Weis, Katharina Schmidt, Jan Heilinger, Jasmin Weindler, Martin Hellmich, Alexander Drzezga, Matthias Schmidt

**Affiliations:** 1 Department of Nuclear Medicine, Faculty of Medicine and University Hospital of Cologne, University of Cologne, Cologne, Germany; 2 German Aerospace Center, Institute of Aerospace Medicine, Cologne, Germany; 3 Institute of Medical Statistics and Computational Biology, Faculty of Medicine and University Hospital of Cologne, University of Cologne, Cologne, Germany; 4 Department of Medical Statistics, University Medical Center Göttingen, Göttingen, Germany

**Keywords:** Low risk thyroid cancer, Papillary thyroid cancer, Follicular thyroid cancer, Radioiodine therapy, survival rate

## Abstract

**Purpose:**

The ESTIMABL2-trial demonstrated non-inferiority of avoiding radioiodine therapy in low-risk differentiated thyroid cancer (DTC, i.e. papillary- and follicular thyroid cancer (PTC, FTC)). Yet, follow-up duration was limited and FTC were underrepresented. We therefore assessed long-term relative survival by comparing observed and expected cancer-free survival. This robust measure of net survival was used to evaluate the long-term effects of radioiodine therapy, including in previously underrepresented histological subgroups.

**Methods:**

Using the SEER-database, we identified 18,645 patients with DTC according to the criteria in the ESTIMABL2-trial (low risk PTC and FTC with pT1am-pT1b, N0-NX). Long-term relative survival (> 10 years) was analysed retrospectively, subdivided based on histology (PTC and FTC) and TNM-status, and additionally in 5,171 patients with lymph node involvement (N1). Relative survival was compared at 3 -, 5 -, and 10-years follow-up with and without radioiodine.

**Results:**

Radioiodine therapy was associated with higher long-term relative survival in specific subgroups: Among FTC patients, relative survival was higher with radioiodine after 5- and, 10-years by 0.3% (*p* = 0.029), and 2.3% (*p* = 0.055), not after three years (0.2%, *p* = 0.150). For PTC without lymph node involvement, no survival difference was detected. With lymph node involvement, relative survival was higher in the radioiodine-group after 10 years (pT1b group with 2.6% difference, *p* = 0.023).

**Conclusion:**

Depending on histology, subgroups of patients with low-risk DTC selected from the SEER-database according to ESTIMABL2-criteria, have a higher survival after radioiodine therapy, notably in FTC patients after more than 5 years. Furthermore, PTC patients with lymph node involvement revealed higher relative survival after 10 years.

**Supplementary Information:**

The online version contains supplementary material available at 10.1007/s00259-026-07888-1.

## Introduction

The American Thyroid Association (ATA) guideline proposed a recurrence risk stratification system using distinct categories for patients with differentiated thyroid cancer (DTC) after initial therapy [1, 2]. Estimated risk of recurrence depends not only on histology (papillary and follicular thyroid cancer (PTC and FTC)) and its subtypes, TNM classification of malignant tumors (TNM), but also discriminates among others between few and a small lymph node metastasis (n ≤ 5 and infiltration depth ≤ 0.2 cm) and invasiveness. A similar risk stratification was proposed by the European Thyroid Association (ETA) with stronger emphasis on TNM [3] and in a recent German guideline [4].

While for high-risk DTC patients the benefit of radioiodine therapy in reducing cancer-recurrence and improving survival is not questioned, in low-risk patients radioiodine is not routinely recommended per ATA and ETA guidelines [1, 2, 5]. Other countries, such as Germany, are more in favor of using radioiodine [4, 6], taking into account the multi-purpose use. Radioiodine not only serves as a treatment option, but also as a sensitive staging tool capable of detecting metastases and remnants with greater sensitivity than conventional non-functional staging methods, such as computed tomography, magnetic resonance imaging, or ultrasonography [4, 7]. As long-term data on survival are scarce and partially conflicting [8, 9], radioiodine therapy remains a matter of controversial debate.

ESTIMABL2, a multicentre randomised phase 3 trial in patients with low-risk DTC (i.e., pT1am or pT1b, N0 or NX, m = multifocal) showed that a treatment without postoperative radioiodine therapy was non-inferior to a treatment involving radioiodine therapy using a low activity of 1.1 GBq I-131 [10, 11]. Lymph node metastases were an exclusion criterion. Recently, an update of the 5-year results showed only a slight trend towards an increase in functional-, structural- or biological abnormalities in the no-radioiodine group compared with the 3-year results with an absolute margin of 2.4% (3-year follow-up: event rate: 4.4% (16/367), 5-year follow-up event rate: 6.8% (24/354)) [11]. The results must be considered in light of the extent of the initial surgery. Remarkably, 86% of patients exhibited an excellent response following surgery alone. This was achieved through total thyroidectomy, as well as the resection of central and/or lateral lymph nodes. A total of 18.8% of patients underwent central lymph node dissection (LND), 17.9% central- and lateral LND, and 6.8% lateral LND. Unlike the standard of clinical practice in many countries, numerous patients underwent extensive surgical procedure without suspicion of lymph node metastasis. Furthermore, the occurrence and number of side effects were not reported despite the fact that more extensive surgery is related to an increase in side effects [12–18]. Moreover, histology subgroups consisted predominantly of PTC. FTC patients were underrepresented with only 3.1% of patients (*n* = 24). Regarding the radioiodine activity, a low activity of 1.1 GBq I-131 was administered. While this is a suitable activity for remnant ablation, it is questionably low for an adjuvant therapy as intended in this context [19]. Thus, long term prognostication and generalization to patient cohorts independent of surgical extent, radioiodine activity, and histologic subtype within the DTC-spectrum remains difficult [19].

With a similar approach compared to the ESTIMABL2 trial, IoN was a multicenter, non-inferiority, phase 3 randomised trial designed to assess whether recurrence-free survival was non-inferior in patients with low-risk DTC who did not receive radioiodine treatment compared with those who did, using the same low radioiodine activity of 1.1 GBq I-131 [20]. DTC patients with pT2, pT3a and N1a were included in addition to the ESTIMABL2 criteria. Nevertheless, the majority of the overall cohort consisted of pT1/pT2 with N0/NX (82.9%, 418/504). The subgroup of all N0/NX patients consisted predominantly of N0 patients (73.4%, 343/467). Similar to ESTIMABL2, histologic subtypes were mostly PTC (78.6%, 396/504) and the median follow-up was slightly longer with 6.8 years. The authors concluded that radioiodine therapy can be avoided in DTC subgroups of pT1, pT2 and N0/NX without affecting recurrence-free survival. Therefore, the results can be regarded as the addition of a pT2 subgroup to the ESTIMABL2 cohort. However, FTC were similarly underrepresented and N0 were overrepresented. Moreover, only 7 patients were under the age of 21 years, in which a potentially more aggressive tumor biology may prevail. Recurrence rates well after 5-year follow-up have previously been reported [21].

Large cancer databases such as the Surveillance, Epidemiology, and End Results Program (SEER) database, provide long-term follow-up survival in patients with DTC with and without radioiodine therapy. Previous database studies showed a trend towards higher long-term overall survival after radioiodine therapy even in low-risk patients. However, inconsistencies with regard to net cancer survival assessed through cancer-specific survival prevailed [8, 9, 22–24]. Recently, a retrospective SEER analysis involving a large number of patients analysed survival relative to the proportion of expected survivors in a comparable set of cancer free individuals (relative survival) as a net survival measure [21]. As opposed to cancer-specific survival, relative survival is inherently not affected by misclassification of death. This in turn is expected to happen more often with older patients and rarer cancer site, as well as slow growing cancers, as in the case of DTC, also corresponding well with the experience gained at our institution. In a series of 15 patients with metastatic DTC who were treated and closely monitored at our institution, and who obviously died due to the direct consequences of metastatic disease, only approximately 20% were correctly classified on the death certificate, while 80% were recorded as having died from causes unrelated to cancer. On the other hand, the relative survival analyses in patients with DTC, inherently not affected by misclassification of death, showed a trend towards higher long-term survival (> 10 years) in certain low-, intermediate and high-risk subgroups, as defined per current guidelines [21]. However, a detailed analysis of an ESTIMABL2-like subgroup including PTC and FTC patients was not performed.

In the following, we used real-world data from the SEER cancer database to analyse the effect of radioiodine therapy on survival of DTC patients who were selected according to the ESTIMABL2 criteria and with lymph node involvement. The analysis included a larger number of patients, including histologic subtypes that were previously underrepresented, and featured a longer follow-up duration.

## Methods

The methodology is similar to [21]. We retrospectively included patients from the SEER-database (SEER Research Plus Data, 17 Registries, Nov 2023) between the years 2000–2021. A total of 194,907 patients (ages > 20 years) harboring thyroid cancer (primary site code “C73 thyroid gland”) were identified. Patients were further divided based on histology and TNM in order to match the definition of low-risk DTC from the ESTIMABL2-trial [10]. We selected patients with classical PTC and FTC and the following TNM were included: pT1am-pT1b, N0-NX (m = multifocal). With a total of 17,981 PTC patients and 664 FTC patients, the ratio of 3.5% harboring FTC resembles in close proximity the ratio of FTC in the ESTIMABL2 trial (3.1%, *n* = 24), at a much larger overall number. Additionally, the non-ESTIMABL2 subgroup of pT1am-pT1b, N1, containing lymph node involvement, was included to further discriminate potential risk factors (*n* = 5171).

To meet the ESTIMABL2 criteria more closely, we excluded all poorly differentiated DTC or aggressive variants, such as “tall cell” or “diffuse sclerosing” PTC, as well as carcinomas with confirmed extrathyroidal extension according to the TNM classification (see supplementary material for further details).

We analysed the composite cohort, but also subgroups based on histology (PTC and FTC) and TNM, in order to elucidate potential effects of radioiodine therapy in clinically relevant subgroups. Lymph node involvement (N1) was additionally analysed.

Survival relative to the proportion of expected survivors in a comparable set of cancer free individuals (relative survival) and, in addition, overall survival and cancer-specific survival including its standard errors were generated using Surveillance Research Program, National Cancer Institute SEER*Stat software (seer.cancer.gov/seerstat) (Version 8.4.3) with the default actuarial method (Similar to [21, 25, 26]). Discrete time intervals of one month up to a maximum follow up time of 220 month were generated. The SEER*Stat default Ederer II method was applied to estimate relative survival as published before [21, 25, 26] (For details see supplemental material). Only cases with confirmed malignancy and matching the SEERs expected survival table were included, identical for overall-, cancer-specific-, and relative survival. As a result, a total of 18,645 in the ESTIMABL2-subgroup and 5171 patients in the N1-subgroups remained.

Most subgroups included a sufficiently large number of patients for an adequate analysis (see Supplementary Table 1, and Table 2 for the exact numbers initially and followed for more than ten years). However, few subgroups were underrepresented. In particular, FTC have a tendency to cause hematogenous metastases instead of lymph node involvement, leading to FTC with N1 to be underrepresented.

Differences between subgroups with and without radioiodine can visually be compared pointwise at any follow-up time as error band are included for each subgroup. For relative survival, the difference between radioiodine and no radioiodine was additionally compared using a SEER default *z*-test developed especially for comparison of relative survival over a pre-defined time period, covering the follow-up intervals of 0–36 month, 0–60 month, and 0–120 month [27].

Despite the fact that relative survival is inherently age-matched to a healthy control group, baseline patient characteristics may still differ with and without radioiodine, given that this database study is not randomised, unlike the ESTIMABL2 trial. We therefore additionally retrieved baseline characteristics from SEER. Table 1 shows an even distribution of patients across subgroups with respect to age, sex and race/ethnicity.

**Table 1:** Baseline Patient characteristics for patients with and without radioiodine therapy (RAI)

		RAI		No RAI	
		n	(%)	n	(%)
total patient number		8758	36.8	15058	63.2
age (years)		49.3 ± 14.6		46.9 ± 13.6	
sex	male	1840	38.6	2924	61.4
	female	6918	36.3	12134	63.7
race/ethnicity	white	5473	36.7	9445	63.3
	black	427	33.8	838	66.2
	American Indian	29	31.5	63	68.5
	Asian or pacific Islander	1087	38.2	1762	61.8
	Hispanic	1675	38.1	2722	61.9

The ethics committee of the University of Cologne approved this retrospective study of cohorts from an anonymized database (reference 24–1168-retro). The requirement to obtain informed consent was waived.

The datasets generated during and/or analysed during the current study are available from the corresponding author on reasonable request.

## Results

### Relative survival not affected by misclassification of cause of death

Following the ideas in a previous SEER-database analysis [21], relative survival was used as an appropriate net survival measure for DTC. Cancer-specific survival and overall survival are included additionally for the composite cohort and PTC subgroups with lymph node involvement for comparison (Supplementary Figs. 1, 2, 3, 4). As previously reported and as expected differences between cancer-specific survival and relative survival appear, with relative survival generally indicating a higher survival rate after radioiodine compared to no-radioiodine subgroups (see for example the FTC subgroup in Supplementary Fig. 1). We attribute these differences to misclassification of cause of death, inherently only affecting cancer-specific survival, which corresponds well to the experience gained in our institution.

### No clinically relevant difference in the composite ESTIMABL2-like cohort

**Fig. 1:**
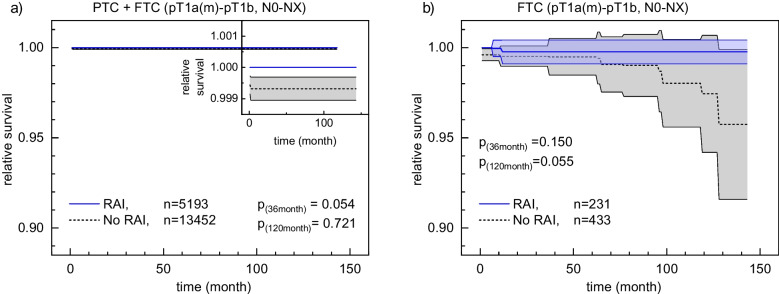
Relative survival including standard-error band in an ESTIMABL2-like cohort with and without radioiodine therapy (RAI) based on the SEER cancer database for **a**) the entire cohort and **b**) the FTC subgroup taken from a). The insert in a) upscales the relative survival measure by an order of magnitude. For the entire cohort, no clinically relevant differences in relative survival appear, consistent with the results from the 5-year follow-up of ESTIMABL2. In contrast for the FTC subgroup a higher 5- and 10-year relative survival after undergoing radioiodine emerges

In Fig. 1 relative survival for low–risk PTC and FTC combined (pT1am-pT1b, N0-NX) (m = multifocal), thus resembling the complete ESTIMABL2 cohort, are depicted with and without radioiodine therapy. In Table 2 differences in relative survival after 3-year, 5-year and 10-year follow-up are given.

**Table 2:** Relative survival (RS) differences with and without radioiodine therapy (RAI) in patients with low-risk differentiated thyroid cancer (i.e. papillary thyroid cancer (PTC) and follicular thyroid cancer (FTC)) according to the definition in the ESTIMABL2 trial including relevant subgroups as well as the non-ESTIMABL2 subgroups with lymph node involvement (N1)

		*n* (beginning)		Absolute difference in 3-year RS: RAI – No RAI (%)	Z (0–3 year)	*p*	Absolute difference in 5-year RS: RAI—No RAI (%)	Z (0–5 year)	*p*	*n* (Follow up > 10 years)		Absolute difference in 10-year RS: RAI—No RAI (%)	Z (0–10 year)	*p*
		RAI	No RAI							RAI	No RAI			
pT1am-pT1b, N0-NX	PTC + FTC	5193	13452	0.07	1.931	0.054	0.07	0.579	0.563	1335	1845	0.07	−0.357	0.721
	PTC	4962	13019	0.06	1.230	0.218	0.06	−0.390	0.697	1278	1790	0.06	−0.880	0.378
	FTC	231	433	0.23	1.439	0.150	0.27	2.180	0.029	57	55	2.33	1.920	0.055
pT1am, N0-NX (m = multifocal)	PTC + FTC	1596	4263	0.04	1.931	0.053	0.04	0.587	0.557	589	1094	0.04	0.159	0.874
	PTC	1586	4250	0.04	1.238	0.216	0.04	−0.399	0.689	586	1088	0.04	−0.889	0.374
	FTC	10	13	7.07	0.828	0.408	7.07	−0.148	0.882	3	6	7.07	0.159	0.874
pT1b, N0-NX	PTC + FTC	3597	9189	0.09	1.520	0.129	0.09	1.190	0.234	746	751	0.09	0.050	0.960
	PTC	3376	8769	0.09	1.087	0.277	0.09	0.767	0.443	692	702	0.09	−0.063	0.949
	FTC	221	420	0.15	1.292	0.196	0.15	2.072	0.038	54	49	2.98	1.864	0.062
pT1am-pT1b, N1 (m = multifocal)	PTC	3560	1605	0.26	−0.289	0.772	0.529	−0.890	0.373	463	153	1.79	0.518	0.604
pT1am, N1 (m = multifocal)	PTC	787	299	0.37	0.019	0.985	0.247	0.539	0.589	214	75	0.89	0.500	0.617
pT1b, N1	PTC	2773	1306	0.39	0.989	0.323	0.555	0.435	0.664	249	78	2.59	2.277	0.023

Differences in RS were tested using a z-test particularly suited for comparing RS

Consistent with the 5-year follow up of ESTIMABL2, no clinically relevant or statistically significant differences between the radioiodine and no-radioiodine groups could be detected (3-year follow-up: *p* = 0.054; 5-year follow-up: *p* = 0.563; and 10-year follow-up: *p* = 0.721).

We therefore proceeded analyzing clinically relevant subgroups in the following.

### Higher relative survival in patients with papillary thyroid cancer with lymph node involvement following radioiodine treatment

**Fig. 2:**
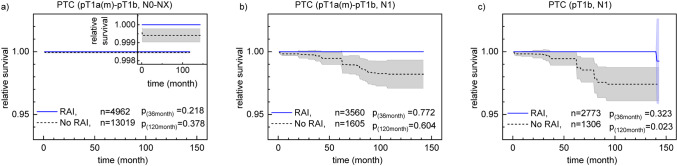
Relative survival including standard-error band with and without radioiodine therapy (RAI) based on the SEER cancer database for **a**) the PTC subgroup of the ESTIMABL2-like cohort, **b**) with the addition of lymph node involvement and **c**) for the pT1b subgroup taken from **b**). Small PTC without verified lymph node involvement **a**) show comparable survival with and without radioiodine. Significantly higher survival after radioiodine appeared if lymph node involvement prevails **b**) and **c**), the higher the larger the tumor size

In Fig. 2 a) relative survival for PTC patients resembling the ESTIMABL2 cohort with and without radioiodine are depicted. Similar to the overall FTC and PTC cohort no clinically relevant and statistically significant difference in 10-year relative survival could be detected (*p* = 0.378) (Table 2). This also holds for any subgroup with regard to T-status (pT1am, N0: *p* = 0.804; pT1b, N0: *p* = 0.741) (see also Supplementary Table 1).

As PTC have a strong tendency to cause lymph node metastasis [4, 28], we additionally compared relative survival for similar T-stages but with clinically relevant verified lymph node involvement (N1) (Table 2, Supplementary Table 1). For multifocal microcarcinoma, 10-year relative survival rate with radioiodine is approximately 1% higher compared to no-radioiodine (Supplementary Fig. 4). Yet, error bars overlap during the entire follow-up and less than 100 patients remaining in the no-radioiodine group after 10 years. Therefore, even if small differences in relative survival were present, statistically significance could not be revealed (10-year follow-up: *p* = 0.617).

For PTC with pT1am-pT1b, N1, relative survival after undergoing radioiodine therapy is approximately 1.8% higher compared to no-radioiodine after a 10-year follow-up. This pointwise survival difference is significantly outside error limits, with error limits starting to spread apart after approximately 6 years. However, the specific z-test for a given relative survival interval did not exhibit significant difference for the 0–120 month interval (*p* = 0.604) (Fig. 2b).

For the PTC subgroup with pT1b, N1 qualitative similar survival curves appeared with larger absolute differences in relative survival. Relevant differences begin to emerge earlier after approximately 4-years follow up, followed by a robust and significantly higher survival after undergoing radioiodine therapy. At 10 years, the relative survival difference reached 2.6% (*p* = 0.023) (Fig. 2c, Table 2).

Relative survival is not impaired in any subgroup undergoing radioiodine therapy compared to the no-radioiodine groups.

### Higher relative survival in patients with follicular thyroid cancer following radioiodine treatment

The ratio of FTC patients compared to the overall number of patients (3.5%) within this study is in close proximity to this ratio in the ESTIMABL2 trial (3.1%).

In Fig. 1b relative survival with and without radioiodine therapy are depicted for the pT1am-pT1b, N0-NX subgroup of FTC patients. Interestingly a clear trend to a higher relative survival rate with radioiodine was observed, which gradually increased during follow up. At 3 years the difference was 0.2% (*p* = 0.150), at 5 years 0.3% (*p* = 0.029), and at 10 years 2.3% (*p* = 0.055), as shown in Table 2. Of note, within this FTC group, the pT1am, N0-NX subgroup (*n* = 23) does not provide meaningful data due to the overall low number of patients (Supplementary Table 1). Therefore, the observed differences were mainly based on significantly higher relative survival rate in the pT1b, N0-NX subgroup.

With FTC generally causing little lymph node involvement, no FTC-N1 analysis provided meaningful results due to low number of patients (Supplementary Table 1).

Relative survival is not impaired after radioiodine therapy compared to not undergoing radioiodine therapy.

## Discussion

The SEER database was used to analyse long-term (> 10 years) relative survival in cohorts defined analogously to the ESTIMABL2-trial [10] with and without radioiodine therapy. No clinically relevant differences were observed for the composite ESTIMABL2-like cohort (i.e. low-risk PTC and FTC with pT1am-pT1b, N0-NX and no aggressive subtypes. Conversely, the FTC-subgroup and PTC with lymph node involvement showed higher relative survival after radioiodine therapy.

Several aspects need to be addressed in order to answer the question which patients in the low-risk group benefit from radioiodine therapy. The SEER database is the most important database to analyse survival in a large number of patients across the broad range of different histologic subtypes in the DTC spectrum with and without radioiodine therapy. In addition to the composite ESTIMABL2 cohort we therefore analysed the PTC- and FTC-subgroups separately.

A total of 18,645 patients in the ESTIMABL2-subgroup in the years 2000 to 2021 were available. As PTC represented the vast majority of the DTC subtypes analysed in both ESTIMABL2 and our study, the survival rate of PTC patients dominantly impacted the overall outcome.

In contrast FTC histology was underrepresented in ESTIMABL2, comprising only 24 patients (3.1%) [10]. In our study, the ratio of FTC to PTC was similar to [11], however at a much larger overall number of patients (*n* = 664, representing 3.6% of the cohort). Higher long-term relative survival after undergoing radioiodine was observed for ESTIMABL2-like FTC (pT1am-pT1b, N0-NX), with differences of 0.3% at 5 years (*p* = 0.029) increasing to 2.3% at 10 years (*p* = 0.055) (Fig. 1b). A long enough follow up time was necessary for the difference to become evident, as they could not be detected after only three years (0.2%, *p* = 0.150) (Table 2). In line with this, a multivariate analysis within the ESTIMABL2 trial revealed that FTC, together with similar oncocytic subtypes, were associated with a higher rate of events indicative of cancer recurrences (Supplementary Table 5 in [11]).

PTC have a strong tendency to cause lymph node involvement. After an unusually extensive surgical approach, lymph node involvement was an exclusion criterion in ESTIMABL2. Therefore, we additionally addressed PTC with verified locoregional lymph node metastasis in 5,171 patients (Fig. 2). The pT1am-pT1b, N1 subgroup showed a clear trend towards higher survival after undergoing radioiodine (pointwise difference at 10 year follow up of approximately 1.8% with difference clearly outside error bands, however, for the entire 0–10 year interval, *p* = 0.604) (Fig. 2b). For pT1b, N1 higher relative survival with radioiodine begins to emerge after approximately 4 years, leading to robust and significant survival difference of 2.6% (*p* = 0.023) after 10-year follow up (Fig. 2c, Table 2).

For multifocal microcarcinoma, only a small number of less than 100 patients were followed for more than ten years. The 10-year relative survival rate with radioiodine is approximately 1% higher compared with no radioiodine, yet error bars overlap (Supplementary Fig. 4, Supplementary Table 1).

When initial surgery includes total thyroidectomy and extended lymph node dissection resulting in complete removal of tumor and no evidence of lymph node involvement present, the addition of radioiodine is unlikely to improve survival in the case of low-risk PTC. However, this radical surgical approach is not a universally accepted strategy. Given the comparably high survival, side effects of surgery are expected to be low in low-risk DTC. ESTIMABL2 did not provide data on laryngeal nerve palsy, hypoparathyroidism, or any other side effects [10, 11].

Moreover in [11] functional, structural, or biological events indicative of cancer recurrence and therefore a measure of progression free survival were reported. A composite outcome strategy was used to define these events. Consequently, each criterion used to define an event, regardless of its potentially differing clinical significance, was weighted equally. Notably, the definition of events was not equal between the radioiodine and no-radioiodine group. For example, uptake outside the thyroid bed could only be measured in patients receiving radioiodine therapy. Biological events included elevated thyroglobulin levels after recombinant human thyrotropin stimulation only in the radioiodine group. For unstimulated thyroglobulin, the cut-off values defining events was 1 ng/mL in the radioiodine therapy group, compared with 2 ng/mL in the no-radioiodine group.

In contrast, SEER predominantly concentrates on the incidence, prevalence, survival, and mortality statistics of cancer. We therefore retrospectively compared long-term relative survival of patients with and without radioiodine therapy, as a robust measure of therapeutic success. Methodologically, this approach does not mirror the event definition in ESTIMABL2 [11]. Nevertheless, as a single outcome measure defined consistently within each subgroup and independent of specific measurement details, it allows for an assessment of survival that incorporates potential consequences of both disease and treatment-related side effects, with the methodological limitations outlined at the end of this section.

The results reported here need to be interpreted in the context of the current era, in which therapy de-escalation is widely discussed, for example, performing lobectomy rather than total thyroidectomy. If only lobectomy is performed, radioiodine therapy is not a treatment option. One possible explanation for the small, yet measurable difference in long-term survival observed here may be initially undetected and therefore untreated metastatic disease. In the case of FTC, undetected vascular invasion may lead to hematogenous spread, potentially resulting in clinically manifest distant metastases. For PTC, hematogenous spread is less common but not impossible, whereas undetected lymph node involvement can occur even in small tumors.

Stable remission after radioiodine therapy can generally be achieved if the absorbed radiation dose per activity to metastatic tissue, in particular distant metastasis, is sufficiently high. This is typically the case when disease volume is relatively small and also depends on the location of metastases [29–31].

Moreover, precise localization of disease is necessary in order to estimate whether radioiodine will achieve remission or if alternative treatment options, such as repeated surgery, are required. As outlined in the introduction, radioiodine serves as a sensitive staging tool. Therefore, if definite and stable long-term survival is the therapeutic goal, achieving remission is a prerequisite, which, in light of these results, would require upfront radioiodine therapy.

On the other hand, as reported in [21] relative and overall survival for most DTC, in particular low-risk DTC, are comparably high. Therefore, patient age and complications of each treatment should be considered. Notably within this study, relative survival was not impaired in any subgroup undergoing radioiodine therapy compared to not undergoing radioiodine-therapy. However, risk of potential side effects of radioiodine-therapy, such as xerostomia, particularly increase after high-activity or recurrent therapies. Additional radioiodine therapies may be associated with increased costs, depending on the clinical course and the available treatment options. On the other hand, if metastases remain undetected after not undergoing radioiodine therapy, surgery for relapse may become necessary, with a greater risk of side effects such as laryngeal nerve palsy.

Our database research has shortcomings. Within SEER we could analyze multifocal microcarcinomas (pT1am) not knowing the sum of the length of the individual of parts. Thus, we did not restrict the total length to > 1 cm and < 2 cm and might have included smaller or larger tumors than in Ref. [10]. Regarding the non-ESTIMABL2 subgroups with lymph node involvement (N1), most guidelines consider few and a small lymph node metastasis (n ≤ 5 and infiltration depth ≤ 0.2 cm) to be less aggressive. In our study, we were able to differentiate only between N0 and N1 status, without information on the exact number of affected nodes or the infiltration depth. As a result, some N1 patients, who would be classified as at least intermediate-risk according to guidelines, may have been included. Nevertheless, this makes it unlikely that the beneficial effect of radioiodine in patients considered low-risk according to the guidelines is overestimated. Additionally, not knowing the exact number of lymph nodes removed during surgery may result in patients being classified as N1 with residual lymph node involvement still being present. After a more radical surgical approach, as performed in ESTIMABL2, such patients might have achieved remission. In this situation, the addition of radioiodine therapy is unlikely to improve survival, at least in cases of PTC, similar to what was stated above for N0 patients. By the same rationale, however, it is also unlikely that any potential beneficial effect of radioiodine is overestimated.

Furthermore, data on the completeness of surgical resection were not available. We nonetheless believe it is unlikely that patients with incomplete resections, who are generally considered at higher risk, would benefit less from radioiodine than those with complete resections.

The radioiodine activity, the number of subsequent radioiodine treatments, details of surgical technique and number of surgeries, as well as biochemical status (thyroglobulin), social status and comorbidities could not be specified, all of which may influence clinical outcome.

In general, SEER-based analyses may be biased by misclassification (e.g., cause of death, as noted above) or data entry errors within the database, representing potential sources of error that are very unlikely to occur in controlled trials.

The retrospective nature is a shortcoming as well.

## Conclusion

In this retrospective SEER-based analysis of low-risk differentiated thyroid cancer cohorts defined according to the criteria in the ESTIMABL2-trial [10, 11] and additionally, with lymph node involvement, radioiodine therapy was associated with higher long-term relative survival in FTC and in PTC with lymph node involvement. Radioiodine therapy should therefore be considered in such subgroups. However, survival differences began to emerge after 5–10 years of follow-up, emphasizing the importance of the length of residual life expectancy for making treatment decisions. No subgroup showed reduced survival after radioiodine therapy.

## Supplementary Information

Below is the link to the electronic supplementary material.

Supplementary file1 (DOCX 1758 KB)

## Data Availability

The datasets generated during and/or analysed during the current study are available from the corresponding author on reasonable request.
