# Supplementary material for: Long-term relative survival with and without radioiodine in patients with low-risk thyroid cancer: a SEER based analysis of histologic subtypes and risk factors
Source: Eur J Nucl Med Mol Imaging. 2026 May 4;53(9):5510–9. doi: 10.1007/s00259-026-07888-1 (PMC13315101; doi:10.1007/s00259-026-07888-1)

**Supplemental Data**

**Method:**

Actuarial method for survival analysis:

Actuarial differs to Kaplan-Meier in the handling of individuals with identical survival times: If deaths occur at the same time as cases are lost to follow-up, the Kaplan-Meier method assumes that all of the cases that were lost to follow-up were at risk at the time of the deaths. The actuarial method assumes that only half of those individuals were at risk at the time of the deaths. Actuarial is therefore suited for applications where data are grouped to time intervals.

Ederer II method for estimating relative survival:

Ederer II calculates expected survival at each point of follow-up such that the matched individuals are considered at risk until the corresponding cancer patient dies or is censored.

Site and morphology codes for stratification according to histology in SEER:

PTC included the following site and morphology codes:

'8050/2: Papillary carcinoma in situ','8050/3: Papillary carcinoma, NOS','8260/2: Papillary adenocarcinoma in situ, NOS','8260/3: Papillary adenocarcinoma, NOS''8340/2: Papillary carcinoma, follicular variant, in situ','8340/3: Papillary carcinoma, follicular variant','8341/2: Papillary microcarcinoma in situ','8341/3: Papillary microcarcinoma','8342/3: Papillary carcinoma, oxyphilic cell','8343/2: Papillary carcinoma, encapsulated, in situ','8343/3: Papillary carcinoma, encapsulated'

FTC included the following site and morphology codes:

'8330/2: Follicular adenocarcinoma in situ','8330/3: Follicular adenocarcinoma, NOS','8331/3: Follicular adenocarcinoma well differentiated','8335/2: Macrofollicular carcinoma in situ','8335/3: Follicular carcinoma, minimally invasive','8339/3: Follicular carcinoma, encapsulated, angioinvasive',

Thus we did not include: any poorly differentiated DTC, tall-cell PTC, clear-cell PTC, diffuse sclerosing PTC, or any anaplastic DTC.

Stage:

The following TNM stages were included: pT1am-pT1b, N0-NX and pT1am-pT1b, N1. Among the aforementioned only T1, T1a, T1b, T1b1 T1a(s), T1b(s) and T1a(m) were retrieved. pT1a2, pT1b2, pT3a2, pT3b, or pT4a were not included, thus tumors with confirmed extrathyroidal extension were not included in the analysis (see for details Schmid KW, Synoracki S, Dralle H, *et al.* Pathologe. 2019;40:18-24, and Deutsche Gesellschaft für Nuklearmedizin (DGN), Deutsche Gesellschaft für Allgemein- und Viszeralchirurgie (DGAV), Deutsche Gesellschaft für Endokrinologie (DGE). S3-Leitlinie Schilddrüsenkarzinom, Version 1.0, July 2025. https://www.leitlinienprogramm-onkologie.de/leitlinien/schilddruesenkarzinom, July 25, 2025 (table 14)).

Inclusion and Exclusion criteria:

We included cases with microscopically confirmed malignancy, known age, and if their first (or only) primary cancer matches all other selection criteria. We excluded cases with missing or unknown values for any expected survival table variable (race, sex, age and date at which the age was coded) in order to match the results to the SEERs expected survival table.

|  |  | n  (beginning) | | Absolute difference in **3 year** RS: RAI - No RAI (%) | Z  (0-3 year) | p | Absolute difference in **5 year** RS: RAI - No RAI (%) | Z  (0-5 year) | p | n  (Follow up > 10 years ) | | Absolute difference in **10 year** RS: RAI - No RAI (%) | Z  (0-10 year) | p |
| --- | --- | --- | --- | --- | --- | --- | --- | --- | --- | --- | --- | --- | --- | --- |
|  |  | RAI | No-RAI |  |  |  |  |  |  | RAI | No Rai |  |  |  |
| pT1am-pT1b, N0 | PTC + FTC | 5100 | 12952 | 0.06 | 1.984 | 0.047 | 0.06 | 0.480 | 0.631 | 1330 | 1812 | 0.06 | -0.420 | 0.674 |
|  | PTC | 4872 | 12550 | 0.05 | 1.270 | 0.204 | 0.05 | -0.582 | 0.561 | 1273 | 1759 | 0.05 | -0.860 | 0.389 |
|  | FTC | 228 | 402 | 0.38 | 1.564 | 0.118 | 0.52 | 2.210 | 0.027 | 57 | 53 | 2.64 | 1.952 | 0.051 |
| pT1am, N0 | PTC + FTC | 1583 | 4212 | 0.04 | 0.838 | 0.405 | 0.04 | -0.093 | 0.926 | 585 | 1077 | 0.04 | 0.238 | 0.812 |
|  | PTC | 1573 | 4199 | 0.04 | 0.846 | 0.398 | 0.04 | -0.076 | 0.939 | 582 | 1071 | 0.04 | 0.248 | 0.804 |
|  | FTC | 10 | 13 | 7.07 | 0.895 | 0.371 | 7.07 | 0.895 | 0.371 | 3 | 6 | 7.07 | 0.895 | 0.371 |
| pT1b, N0 | PTC + FTC | 3517 | 8740 | 0.09 | 1.178 | 0.239 | 0.09 | 0.350 | 0.726 | 745 | 735 | 0.09 | -0.227 | 0.820 |
|  | PTC | 3299 | 8351 | 0.07 | 0.680 | 0.497 | 0.07 | 0.440 | 0.659 | 691 | 688 | 0.07 | -0.330 | 0.741 |
|  | FTC | 218 | 389 | 0.18 | 1.390 | 0.165 | 0.34 | 1.990 | 0.047 | 54 | 47 | 3.36 | 1.890 | 0.058 |
| pT1am-pT1b, NX | PTC + FTC | 93 | 500 | 0.31 | 1.264 | 0.206 | -0.43 | 0.571 | 0.568 | 5 | 33 | -3.29 | 0.262 | 0.793 |
|  | PTC | 90 | 469 | 0.37 | 1.131 | 0.258 | -0.36 | 0.510 | 0.610 | 5 | 31 | -3.22 | 0.254 | 0.799 |
|  | FTC | 3 | 31 | n/a | n/a | n/a | n/a | n/a | n/a | 0 | 2 | n/a | n/a | n/a |
| pT1am, NX | PTC + FTC | 13 | 51 | 0 | 0 | 0 | -6.08 | -1.898 | 0.058 | 4 | 17 | -13.19 | -2.720 | 0.007 |
|  | PTC | 13 | 51 | 0 | 0 | 0 | -6.08 | -1.897 | 0.058 | 4 | 17 | 13,19 | -2.720 | 0.007 |
|  | FTC | 0 | 0 | n/a | n/a | n/a | n/a | n/a | n/a | 0 | 0 | n/a | n/a | n/a |
| pT1b, NX | PTC + FTC | 80 | 449 | 0.39 | 1.245 | 0.213 | 1.75 | 1.408 | 0.159 | 1 | 16 | 6,04 | 1.643 | 0.100 |
|  | PTC | 77 | 418 | 0.56 | 1.300 | 0.194 | 0.66 | 1.394 | 0.163 | 1 | 14 | 7,64 | 1.700 | 0.089 |
|  | FTC | 3 | 31 | 0 | n/a | n/a | n/a | n/a | n/a | n/a | 2 | n/a | n/a | n/a |
| pT1am-pT1b, N1 | FTC | 5 | 1 | n/a | n/a | n/a | n/a | n/a | n/a | 2 | 1 | n/a | n/a | n/a |
| pT1am, N1 | FTC | 1 | 0 | n/a | n/a | n/a | n/a | n/a | n/a | 1 | 0 | n/a | n/a | n/a |
| pT1b, N1 | FTC | 4 | 1 | n/a | n/a | n/a | n/a | n/a | n/a | 1 | 1 | n/a | n/a | n/a |

**Supplementary Table 1**: Relative survival (RS) differences with and without radioiodine therapy (RAI) in patients with low risk differentiated thyroid cancer (i.e. papillary thyroid cancer (PTC) and follicular thyroid cancer (FTC)) in subgroups of cohorts resembling the definition in the ESTIMABL2 trial as well as the non- ESTIMABL2 subgroups with lymph node involvement (N1). Differences in RS were tested using a z-test particularly suited for comparing RS.





**Supplementary Figure 1**: Relative survival, cancer-specific survival, and overall survival including standard-error band for patients with low-risk differentiated thyroid cancer resembling the definition from the ESTIMABL2 trial. Top row: entire cohort, middle row: papillary thyroid cancer (PTC) subgroup, and bottom row: follicular thyroid cancer (FTC) subgroup.


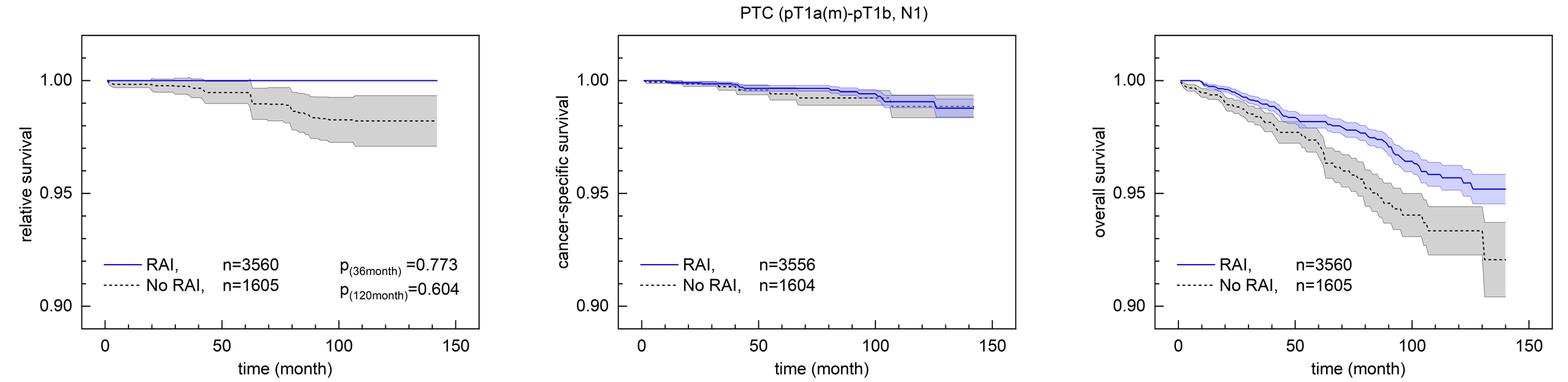


**Supplementary Figure 2**: Relative survival, cancer-specific survival, and overall survival in a cohort of patients with papillary thyroid cancer (PTC) harboring lymph node involvement (N1) but otherwise identical to the cohort in Supplementary figure 1 (resembling the ESTIMABL2 trial).


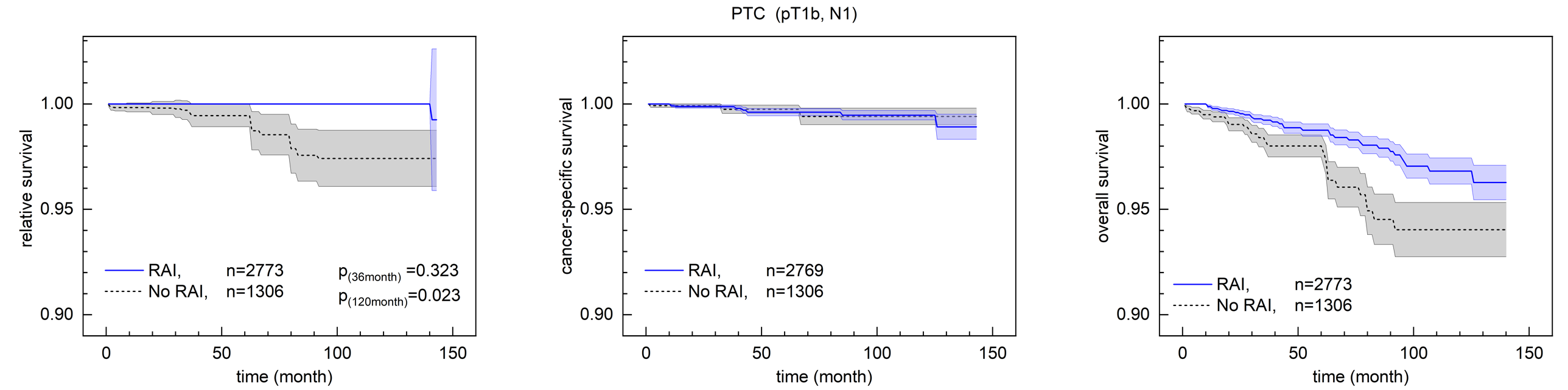


**Supplementary Figure 3**: Relative survival, cancer-specific survival, and overall survival in a pT1b cohort of patients with papillary thyroid cancer (PTC) harboring lymph node involvement (N1) but otherwise identical to the cohort in Supplementary figure 2 (resembling the ESTIMABL2 trial).

***Supplementary Figure 4****: Relative survival, cancer-specific survival, and overall survival in a pT1a(m) cohort of patients with papillary thyroid cancer (PTC) harboring lymph node involvement (N1) but otherwise identical to the cohort in Supplementary figure 2 (resembling the ESTIMABL2 trial).*


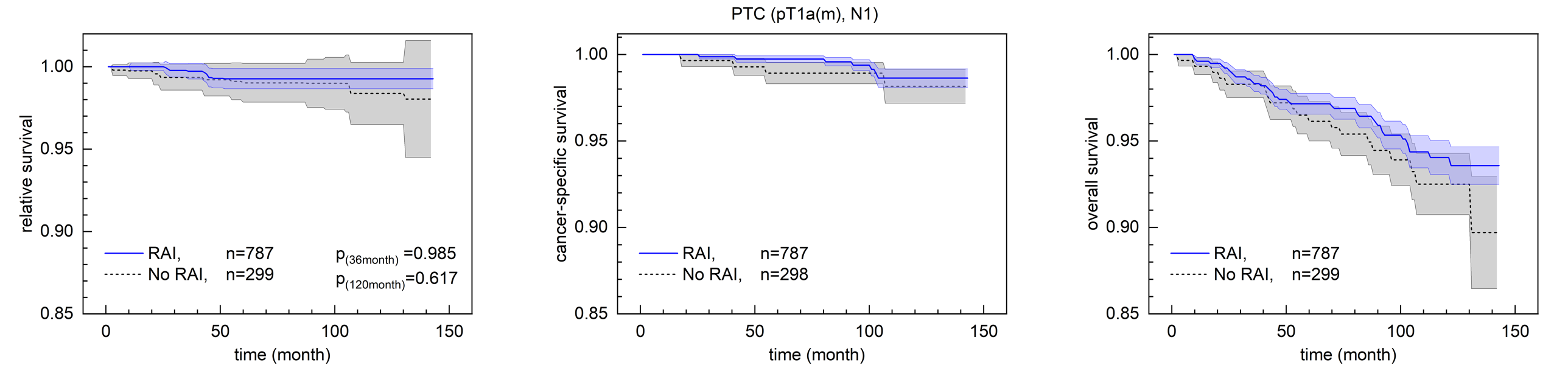

Supplement: Supplementary file 1 — Supplementary file1 (DOCX 1758 KB) [file 259_2026_7888_MOESM1_ESM.docx]
